# Supplementary material for: Tracking calcium dynamics from individual neurons in behaving animals
Source: PLoS Comput Biol. 2021 Oct 8;17(10):e1009432. doi: 10.1371/journal.pcbi.1009432 (PMC8528277; doi:10.1371/journal.pcbi.1009432)
Supplement: Attachment — Submitted filename: review_PCB_Lagache_2020.pdf [file pcbi.1009432.s003.pdf]

This paper entitled “Robust tracking of single neuron calcium dynamics in behaving Hydra” presents EMC2, a new image analysis pipeline for the tracking of intermittent objects in a deforming reference medium. Tracking cells in complex environments, either deforming or crowded, is a very important technique applied way beyond the field of neuroscience but which suffers from well-known limitations and caveats. The paper clearly demonstrates that the EMC2 algorithm outperforms state of the art methods in the specific case of calcium based neuronal tracking in Hydra.

The authors combine several existing tools with a truly original step which consists in using tracktable cells to compute the elastic deformation of the reference medium (e.g. the Hydra body). This deformation is then used to infer the position of cells at time points at which they are not detectable. This step allows to concatenate multiple short tracks into long ones based on the expected deformation of the reference medium. The usefulness of this approach is clearly demonstrated on that test case and the implementation of the algorithm in an open-source platform could be a great asset.

However, as a mostly technical paper, its main limitation is the lack of other test cases or at least a discussion of its domain of applicability. Along the same line, a discussion/demonstration of the limits of this technique would be very valuable. The absence of such discussions and the focus on the Hydra nervous system limit the potential readership of this paper which is of a very good scientific quality and could, in my opinion, have an impact much broader than Hydra calcium imaging. I fear that in its current form, this paper would go largely unnoticed by people outside of the Hydra or neuro-imaging communities.

Major comments:

1- As described by the authors, EMC2 is designed to address at the same time two difficult aspects of cell tracking: interrupted tracks due to improper/impossible detection and the deformation of the reference medium. Although calcium based (hence intermittent) neuronal tracking in Hydra (hence deforming) is a perfect example of such a case, one sees that this technique was specifically developed for that problem and that the synthetic data also resembles that test case. The authors discuss in the Introduction the use of small animals for the imaging of full nervous systems but fail to show or at least discuss whether this technique would also be applicable to these organisms. The reader is therefore left with the question of whether this is a Hydra specific technique or if it is useful for the entire field of calcium imaging in small animals. Furthermore, the two difficulties this algorithm tackles can be encountered in other situations than calcium imaging, for instance in the propagation of a dense front of cells or the collective migration of a confluent tissue. Demonstrating the applicability of EMC2 in these different situations, through technical arguments or even examples based on available data, would greatly enhance the impact of the paper.
2- As this is a mostly technical paper, the presentation of the procedure, some of its limitations and the expected outputs lack details in some parts. Specific examples will be described first below.

Major revisions:

1- ‘ ECM2 algorithm’ section: description of how the stitching of short tracks works should be more detailed. For instance, it is not clear whether all short tracks will be stitched or if some

will be lost. From reading the Methods section, I imagine that some tracks will be lost. Discussion of how many and which would be interesting. Similarly, the quantitative comparisons of Fig3 do not show whether EMC2 is more efficient because most of its tracks are correct regardless of how many or because it achieves reconstruction of more tracks than other algorithms.

2- It seems that a key to the robustness of EMC2 is to have a sufficient number of “stable cells” in order to properly infer the elastic deformation of the medium. This is achieved in Hydra by the presence of transfected nematocytes and in synthetic data by the application of 20% of stable cells. This seems to be a possible important limitation of the technique. Would that be a problem for the application of EMC2 to other systems? What fraction of stable cells is required to ensure robustness of the technique? A more thorough study of the impact of alpha_stable in synthetic data would greatly strengthen the claim that this method is robust. A more thorough discussion of this aspect, the limitations it implies and the cases where this is possible could help broaden the impact and diffusion of the technique.

3- The expected output of the algorithm is not entirely clear. Fig1-6 seems to imply that one should expect a list of tracks spanning the entire length of the movie. However, from the Methods section, it seems possible that a short track can’t be concatenated with others. Is such a track eliminated entirely? Can the output contain partial tracks? In relation to this, since the elastic deformation is propagated forward only: is the number of expected tracks directly dependent on the number of cells detected at the first time step? Clarifications on this topic would make the technique more comprehensible and more directly applicable. Modifications of Fig 1-6 to show partial tracks would also clarify this point.

Minor revisions:

1- I believe it would be worth mentioning in the title that the paper presents a new algorithmic technique for automated tracking. The focus on the Hydra test case could impair the visibility of the paper for some target communities.

2- Similarly, I would suggest changing the keywords assigned to the paper to better reflect the fact that it presents a new tracking algorithm for intermittent signals and/or deforming media. This would help the dissemination of the technique to other fields.

3- In several places, the authors mention the importance of calcium imaging in freely behaving animals. In the test case, the Hydras are still confined by a coverslip even though they do display deformations and normal behavior. Still, this should be mentioned clearly in the main text and care should be taken not to claim this example as a “freely behaving animal”.

4- In Fig 1-5, the meaning of the arrows is not clear. I imagine they show the “decisions” that have to be made to concatenate short tracks but, as presented, these arrows seem mostly random.

5- Between p8 and p9, the authors claim that “EMC2 contains only one free parameter” which is described as d_max but Methods also introduce gap_max as a second free parameter. Clarification is needed on this point.

6- As described above, information on the number of obtained tracks is given for the tracking of Hydra neurons but not for the synthetic data. This should be added either in Fig3 or in the corresponding text.

7- In Fig4, a figure showing at least one example of silhouette scores versus number of clusters would be nice to support the claim that “We found 2 or 3 neuronal ensembles in each movie”.

8- In this section and at the end of the discussion, the authors claim that the neuronal ensembles are “non-overlapping”. Although this was previously demonstrated, this claim is not supported by data in this paper. Precision on the fact that no, or very few, neurons were found to belong to two different ensembles (i.e. firing in more than 30% of ensemble spikes) would suffice to support this claim.

9- In fig4-f, it seems that the three ensembles come from the same experiment (3rd line in Table3) but the three pictures are different. Wouldn’t the case be made even stronger if the three ensembles were labeled in a single image?

10- Figure subpanels labelling should be added to Fig3 and homogenized between figures.

11- In the last paragraph of the discussion section, the authors claim that “EMC2 is a robust and versatile tracking algorithm”. This claim of versatility needs to be further supported by discussion or demonstration of its applicability to different situations.

12- In Material and Methods, section 1, the explanation of the assignment problem that concatenates tracks is not very detailed. In particular, the constraints applied to i and j are not clear. What if a track has no other track within the d_max distance? What if concatenation doesn’t lead to full tracks?

13- I have never used Icy before but tried to apply EMC2 to my own data on this platform. The code works but using it is not straightforward especially for people not used to programing. Time allowing, a more detailed, step-by-step protocol would most likely greatly increase the dissemination of the technique.
