# Supplementary material for: Tracking calcium dynamics from individual neurons in behaving animals
Source: PLoS Comput Biol. 2021 Oct 8;17(10):e1009432. doi: 10.1371/journal.pcbi.1009432 (PMC8528277; doi:10.1371/journal.pcbi.1009432)
Supplement: Attachment — Submitted filename: Response to reviewers.docx [file pcbi.1009432.s004.docx]

Thursday, February 11^th^, 2021

Dear Professor Gutkin,

We are submitting our revised manuscript entitled " *EMC^2^: A versatile algorithm for robust tracking of calcium dynamics from individual neurons in behaving animals*" for your consideration as an Article in PLOS Computational Biology. We would like to thank you for giving us the opportunity to respond to the reviewers’ concerns.

To answer Reviewers main concerns, we have now:

1- **Discussed the versatility of our tracking method, and showed its potential application to other model organisms**. In particular, we used EMC^2^ to track single neurons in two-photon imaging of mouse visual cortex (new Figure 4 and Result section 4-a in the revised manuscript). Even if tracking the limited stochastic motion of neurons in head-fixed animals is much less challenging than tracking neurons in highly deforming animals like *Hydra*, this second application shows the capacity of our algorithm to track neurons in various experimental systems. We have also changed the title to “*EMC^2^: A versatile algorithm for robust tracking of calcium dynamics from individual neurons in behaving animals*”, and we have discussed the possible extension of EMC^2^ to three-dimensional imaging of other animal models.
2- **Added details about the stitching procedure between short *tracklets*** initially produced by the probabilistic tracking algorithm.
3- **Tested the robustness of EMC^2^ for a large class of simulation parameters**, including the percentage of stable (i.e. non-blinking) particles, the number (density) of simulated particles and the length of simulated time-lapse sequences (new Figure 3 d,e&f). We showed that, while the number of particles can affect tracking performances, EMC^2^ was very robust to the percentage of stable cells and the length of simulated sequences.
4- **Clarified the novelty of our approach, especially compared to the NeRVE method of Nguyen et al. [1].** NeRVE has been designed for the tracking of neurons in *C.Elegans* labeled with calcium-insensitive probes, after having “*straightened*” the worm and mapped neuron coordinates along the principal axis of the animal. This method, based on point-set registration and global clustering of neuron coordinates, is not sufficiently robust for tracking intermittent signal of neuron calcium imaging in highly deforming environments like *Hydra*, as we previously showed in [2]. Therefore, we have designed EMC^2^ as a hybrid approach combining the accuracy of probabilistic tracking of neurons when they are detectable, and the iterative estimation of animal deformation followed by local *tracklet* concatenation.

Looking forward to a favorable response

Yours sincerely,

Thibault Lagache and Rafael Yuste

**Reviewer #1:**

This paper entitled “Robust tracking of single neuron calcium dynamics in behaving Hydra” presents EMC2, a new image analysis pipeline for the tracking of intermittent objects in a deforming reference medium. Tracking cells in complex environments, either deforming or crowded, is a very important technique applied way beyond the field of neuroscience but which suffers from well-known limitations and caveats. The paper clearly demonstrates that the EMC2 algorithm outperforms state of the art methods in the specific case of calcium based neuronal tracking in Hydra.

The authors combine several existing tools with a truly original step which consists in using tracktable cells to compute the elastic deformation of the reference medium (e.g. the Hydra body). This deformation is then used to infer the position of cells at time points at which they are not detectable. This step allows to concatenate multiple short tracks into long ones based on the expected deformation of the reference medium. The usefulness of this approach is clearly demonstrated on that test case and the implementation of the algorithm in an open-source platform could be a great asset.

However, as a mostly technical paper, its main limitation is the lack of other test cases or at least a discussion of its domain of applicability. Along the same line, a discussion/demonstration of the limits of this technique would be very valuable. The absence of such discussions and the focus on the Hydra nervous system limit the potential readership of this paper which is of a very good scientific quality and could, in my opinion, have an impact much broader than Hydra calcium imaging. I fear that in its current form, this paper would go largely unnoticed by people outside of the Hydra or neuro-imaging communities.

***Major comments:***

1- As described by the authors, EMC2 is designed to address at the same time two difficult aspects of cell tracking: interrupted tracks due to improper/impossible detection and the deformation of the reference medium. Although calcium based (hence intermittent) neuronal tracking in Hydra (hence deforming) is a perfect example of such a case, one sees that this technique was specifically developed for that problem and that the synthetic data also resembles that test case. The authors discuss in the Introduction the use of small animals for the imaging of full nervous systems but fail to show or at least discuss whether this technique would also be applicable to these organisms. The reader is therefore left with the question of whether this is a Hydra specific technique or if it is useful for the entire field of calcium imaging in small animals. Furthermore, the two difficulties this algorithm tackles can be encountered in other situations than calcium imaging, for instance in the propagation of a dense front of cells or the collective migration of a confluent tissue. Demonstrating the applicability of EMC2 in these different situations, through technical arguments or even examples based on available data, would greatly enhance the impact of the paper.

**Answer:** We thank the reviewer for having pointed out that our method is, indeed, a general tracking procedure that can be applied to other, if not all, model organisms and could eventually be applied to other tracking problems such as cellular tracking in confluent tissues during morphogenesis or wound healing.

We agree that in the previous version of the manuscript, the applicability of our method to other organisms was not clearly demonstrated, or at least discussed. We have now revised the manuscript to highlight the versatility of our method:

i. We have changed the title to: “*EMC^2^: A versatile algorithm for robust tracking of calcium dynamics from individual neurons in behaving animals*”
ii. We have added a paragraph in the Introduction section to clearly state that our tracking method is in line with several hardware and software developments in the past few years, that aimed at robustly tracking neurons in many model organisms:

“*To tackle these issues, numerous hardware solutions have been proposed, including the fixation of animal (head) [3-5], the high-speed motorization of microscopes to track animal movements [6, 7] and the dual-color labeling of neurons with a calcium insensitive probe that can be detected and localized even when neurons are not firing [7, 8]. However, even when (some of) these hardware solutions are implemented, the residual motion of neurons, the limited spatial resolution of the microscope and the intermittency of fluorescence signal (when single-channel calcium imaging is used) hinder the robust tracking of single neuron activity. Therefore, elaborated post-processing of acquired movies is required. Most of developed image processing methods consist of registering images (volumes) to reference image(s) (volume(s)) using either the local fluorescence intensity of images [9, 10], or directly the extracted neuron positions [1, 2, 7, 11]. Then, additional image processing, such as non-negative matrix factorization [12, 13], might be required for demixing and denoising cellular calcium activity.*

*Despite all these software developments, the difficult implementation of hardware solutions, such as the dual-labeling and imaging of calcium-insensitive probes, together with the important deformability of challenging animal models such as Hydra or Caenorhabditis elegans, prevent the robust automatic tracking of single neurons in many experiments. Tracking has then to be performed manually [14] or semi-manually [15].*”

iii. In addition to the challenging *Hydra* model, we have used the EMC^2^ method to track single neurons in two-photon imaging of mouse visual cortex. Two-photon imaging is widely used to monitor neuronal activity in targeted brain regions of small animals [5], and even if the head of the animal is fixed under the microscope, the residual motion of neurons call for image post-processing (image registration and neuronal cell mask segmentation typically [10, 12]) to accurately measure single cell activity. We showed that EMC^2^ is an automatic, fast and robust tracking-based alternative to traditional image processing. Our results are summarized in the Results section 4-a “*Monitoring neuron activity with two-photon microscopy in mouse cortex*” and new Figure 4 (reproduced hereafter).

In (head-) fixed animals, single neuron trajectories can be modeled with stochastic confined motion, that are much more trivial to track than neurons in animal models that experience major deformations like *C. Elegans* or *Hydra*. Therefore, the validation of EMC^2^ in these two extreme scenario (Mouse and *Hydra*) show that our method is sufficiently versatile to be applied in most, if not all, animal models.

iv. For the time being, EMC^2^ algorithm is only implemented for tracking two-dimension movies. We have now added a paragraph in the Discussion section to discuss the possible (and easy) extension of our algorithm to three-dimension movies, allowing neuron tracking in a larger class of animal models where three-dimensional high-speed imaging is routinely used [3, 8]:

“*On-going development of high-speed three-dimensional microscopes enable the imaging of neuronal activity with high temporal and spatial resolution in an increasing number of animal models [3, 8]. In a near future, the extension of EMC^2^ tracking method to three-dimension movies should not present important technical issues. Indeed, the thin-plate-spline transform used here to estimate the deformation of the field-of-view in two-dimensions is actually a special case of polyharmonic splines that have been specifically designed for the robust interpolation between data points in any dimensional space [16]. “*

***Figure 4: Monitoring calcium activity from individual neurons with two-photon calcium imaging of mouse visual cortex and EMC^2^.*** *a- Two-photon imaging of calcium activity from individual neurons in visual cortex of awake mice is performed at Day 1, Day 2 and Day 46 during 5 minutes at 12.3 Hz. Tracking of neuron positions is performed with EMC^2^ and reveals an important turn-over of active neurons across days. Examples of neurons that are active at Day 1, Day 2 or Day 46 are respectively highlighted with red, green or blue arrows. Neurons active at Day 1&2 are highlighted with yellow arrows, at Day 2&46 with cyan arrows, and at Day 1,2&46 with white arrows. The median number of active neurons each day is also plotted (n=4 animals). The number of neurons that are active from Day 1, 2 or 3 are respectively represented in red, green or blue. b- Single neuron trajectories can be modeled with confined stochastic motion. Two example trajectories are shown (green & blue trajectories) with a maximum excursion distance of ~ 4 pixels. Boxplots of single neuron displacement between two consecutive frames, and maximum excursion distance (in pixels) are plotted (n=590 trajectories).*

2- As this is a mostly technical paper, the presentation of the procedure, some of its limitations and the expected outputs lack details in some parts. Specific examples will be described first below.

***Major revisions:***

1- ‘ ECM2 algorithm’ section: description of how the stitching of short tracks works should be more detailed. For instance, it is not clear whether all short tracks will be stitched or if some will be lost. From reading the Methods section, I imagine that some tracks will be lost. Discussion of how many and which would be interesting.

**Answer:** After having estimated iteratively the forward (resp. backward) position of particles after the ending point (resp. before the starting point) of each short track (tracklet), we concatenated tracklets that **1-** could be concatenated (i.e. whose propagated estimations of positions *“meet”* at some time point, i.e. are closer than a user-defined maximum distance) and **2-** that minimize the overall sum of distances between all concatenated tracklets (linear assignment problem).

Therefore, each tracklet is not necessarily stitched (i.e. concatenated) to another tracklet, and final *long* tracks, that putatively correspond to entire trajectories of single particles, are resulting from the concatenation of $n\geq1$ tracklet(s). We highlight that each *long* track does not necessarily span the entire time lapse sequence: it starts with the starting-point of its first concatenated tracklet, and ends with the ending-point of its last concatenated tracklet.

We have now added details about the stitching (concatenation) step in the paragraph ***“Elastic motion correction and concatenation (EMC2) of short tracks”*** of the Methods section:

*“The eMHT algorithm returns a set of N short tracks (tracklets)* $\tau_{i}, for 1\leq i\leq N$*, of detectable particles; the ith tracklet* $\tau_{i}$*starts at position* $\boldsymbol{x}_{i}(t_{i}^{s})$ *at time* $t_{i}^{s}$ *and ends at position* $\boldsymbol{x}_{i}(t_{i}^{e})$ *at time* $t_{i}^{e}$*. To concatenate tracklets, i.e. to link the tracklets that putatively correspond to the same neuron, we then estimate the backward position* ${\hat{\boldsymbol{x}}}_{\boldsymbol{i}}^{\boldsymbol{b}}(t)$ *of the particle (neuron) corresponding to the* $i^{th}$ *tracklet before the starting-point (*$t\leq t_{i}^{s}$*) of the tracklet. To estimate* ${\hat{\boldsymbol{x}}}_{\boldsymbol{i}}^{\boldsymbol{b}}(t)(t)$ *for* $t\leq t_{i}^{s}$*, we iteratively apply backward thin-plate-spline transformation* ${\hat{\boldsymbol{x}}}_{\boldsymbol{i}}^{\boldsymbol{b}}\left( t-1 \right)=TPS_{backward}({\hat{\boldsymbol{x}}}_{\boldsymbol{i}}^{\boldsymbol{b}}(t))$*, with the initial condition* ${\hat{\boldsymbol{x}}}_{\boldsymbol{i}}^{\boldsymbol{b}}\left( t_{i}^{s} \right)=\boldsymbol{x}_{i}(t_{i}^{s})$*. Similarly, to estimate the forward position* ${\hat{\boldsymbol{x}}}_{\boldsymbol{i}}^{\boldsymbol{f}}(t)$ *after the ending-point of the tracklet, i.e. for* $t\geq t_{i}^{e}$*, we iteratively apply the forward transformation* ${\hat{\boldsymbol{x}}}_{\boldsymbol{i}}^{\boldsymbol{f}}\left( t \right)=TPS_{forward}({\hat{\boldsymbol{x}}}_{\boldsymbol{i}}^{\boldsymbol{f}}(t-1))$ *with the initial condition* ${\hat{\boldsymbol{x}}}_{\boldsymbol{i}}^{\boldsymbol{f}}\left( t_{i}^{e} \right)=\boldsymbol{x}_{\boldsymbol{i}}\left( t_{i}^{e} \right).$ *From the estimated positions of particles corresponding to each tracklet, we then compute the distance* $d_{i,j}$ *between tracklets* $\tau_{i}\neq\tau_{j}$ *with*

$$d_{i,j}=\left\{ \begin{aligned} \infty, if t_{i}^{e}>t_{j}^{s} or t_{j}^{e}>t_{i}^{s} \\ \min_{t_{i}^{e}\leq t\leq t_{j}^{s}} \left\| {[\hat{\boldsymbol{x}}}_{i}^{f}-{\hat{\boldsymbol{x}}}_{j}^{b}]\left( t \right) \right\|, if t_{i}^{e}\leq t_{j}^{s}\leq t_{i}^{e}+gap_{max} \\ \min_{t_{j}^{e}\leq t\leq t_{i}^{s}} \left\| {[\hat{\boldsymbol{x}}}_{j}^{f}-{\hat{\boldsymbol{x}}}_{i}^{b}]\left( t \right) \right\|, if t_{j}^{e}\leq t_{i}^{s}\leq t_{j}^{e}+gap_{max} \end{aligned} \right.$$

*where* $gap_{max}$ *is a user-defined maximum time gap that EMC2 is allowed to close (typically a few hundreds of frames). We need to apply a maximum time gap for time-lapse sequences with particles that remain undetectable over long period of time due to the error growth during backward/forward estimation of the putative position of undetectable particles.*

*Using the computed distances between tracklets, we then define an association cost matrix,*

$$\boldsymbol{\Phi}= \left( \begin{matrix} \phi_{1,1} & \cdots& \phi_{1,N} \\ \vdots& \ddots& \vdots\\ \phi_{N,1} & \cdots& \phi_{N,N} \end{matrix} \right),$$

*with* $\phi_{i,j}=d_{i,j}$ *if* $d_{i,j}<d_{max}$*, and* $\phi_{i,j}=\infty$ *otherwise.* $d_{max}$ *is the second user-defined parameter of our tracking algorithm that specifies the maximum distance allowed between the forward-propagated end-point of a tracklet and the backward-propagated starting-point of another tracklet. Finally, among all possible associations for which the cost* $\phi_{i,j}<\infty$*, the optimum set of concatenated tracklets* $\tau_{i^{*}}\to\tau_{j^{*}}$ *among all the tracklets* $\left\{ \tau_{i},\tau_{j} \right\}, 1\leq i,j\leq N$ *is the solution of the global minimization problem*

$$i^{*}\to j^{*}=\min_{{i,j such that \phi}_{i,j}<\infty} \sum\phi_{i,j}.$$

*This minimization problem, known as the assignment problem, is similar to the problem solved in GDM methods of tracking, where algorithms determine the optimal association between particle detections by minimizing the global distance between detections in consecutive time frames of the sequence. One of the first and most popular algorithms to solve assignment problems is the Hungarian algorithm [17]. However, due to its computational load, faster algorithms have been proposed over the years. We used here the Jonker-Volgenant algorithm [18] implemented in the TrackMate plugin in ImageJ (see table 1).*

*Finally each particle (neuron) track* $T_{i}$ *for* $1\leq i\leq N^{'}$*, with* $N^{'}\leq N$ *the number of “long” tracks, results from the concatenation of* $n_{i}$ *tracklets:* $T_{i}=\tau_{i_{1}}\to\tau_{i_{2}}\to\ldots\to\tau_{i_{n_{i}}}$*with* $t_{i_{1}}^{e}\leq t_{i_{2}}^{s}\leq\ldots\leq t_{i_{n_{i}}}^{s}$ *. We highlight that, by construction, each long track* $T_{i}$ *does not necessarily span over the whole time-lapse imaging sequence, but begins at the starting-time* $t_{i_{1}}^{s}$ *of the first tracklet* $\tau_{i}$ *and ends at the ending-time* $t_{i_{n_{i}}}^{e}$ *of the last tracklet* $\tau_{i_{n_{i}}}$ *.”*

Similarly, the quantitative comparisons of Fig3 do not show whether EMC2 is more efficient because most of its tracks are correct regardless of how many or because it achieves reconstruction of more tracks than other algorithms.

**Answer:** To show that the high accuracy of EMC^2^ algorithm is not associated with an over-number of reconstructed tracks, we have now indicated the number of simulated and reconstructed tracks for each simulation throughout the manuscript.

2- It seems that a key to the robustness of EMC2 is to have a sufficient number of “stable cells” in order to properly infer the elastic deformation of the medium. This is achieved in Hydra by the presence of transfected nematocytes and in synthetic data by the application of 20% of stable cells. This seems to be a possible important limitation of the technique. Would that be a problem for the application of EMC2 to other systems? What fraction of stable cells is required to ensure robustness of the technique? A more thorough study of the impact of alpha_stable in synthetic data would greatly strengthen the claim that this method is robust. A more thorough discussion of this aspect, the limitations it implies and the cases where this is possible could help broaden the impact and diffusion of the technique.

**Answer:** We have now measured the sensitivity of EMC^2^ to several parameters: the percentage of stable (i.e. non-blinking) particles, the number (density) of particles and the length of simulated time-lapse sequences (new Figure 3-d,e&f reproduced below). Overall, these simulations show that EMC^2^ is robust to the percentage of stable particles and the increasing length of simulated sequence, but rapidly deteriorates as the number of particles (actually their density) decreases. Indeed, in simulations with very few simulated particles, the forward and backward deformation of the field-of-view cannot be accurately computed due to the lack of fiducial points, leading to important error propagation and erroneous tracklet concatenation.

*“Figure 3:* ***Testing EMC^2^ robustness with synthetic simulations.****…(d-e) Using “Hydra-like” synthetic deformation, we measured the accuracy of EMC2 for increasing proportion of stable (i.e. non-blinking) particles (neuronal cells) and increasing number of simulated particles. (f) After having estimated the deformation-field in three different animals (animal 1 (red), 2 (orange) and 6 (green)), we measured the accuracy of EMC2 for simulated sequences with increasing length (250, 500 and 900 or 1000 frames).”*

We have also added the following paragraphs in the Result section 3.b “Synthetic time-laspse sequences” of the manuscript:

*First, we found that even without stable cells (*$\alpha_{stable}=0$*), the accuracy of EMC^2^ remained high (77.7 %* $\pm$ *3.6% (398.8* $\pm$ *18.4 correct tracks over 513.3* $\pm$ *1.7 reconstructed tracks), and rapidly increased to 91.8 %* $\pm$ *1.2% for* $\alpha_{stable}=5\%$*, before reaching a plateau above 95% accuracy for* $\alpha_{stable}\geq10\%$*. Conversely, we found that EMC^2^ was very sensitive to the number of neurons, with poor performance for very few neurons (for simulations with only 10 neurons, the accuracy was 11.6 %* $\pm$ *0.6% (5.6* $\pm$ *0.3 correct tracks over 49.8* $\pm$ *0.8 reconstructed tracks)). The accuracy rapidly increased to > 90% when more than 100 neurons were simulated. Tracking errors in simulations with few neurons are due to the inaccurate estimation of the deformation field, and iterative error propagation, when few fiducial source and target points are used. Finally, we measured how the performance of the EMC^2^ decreased with the length of the simulated sequence, for three different animals (animal 1, 2 and 6 see Table 3). We chose animals with highest number of neurons so that we could accurately estimate, and therefore simulate, their body deformation. We found that the EMC^2^ accuracy slowly decreased with the sequence length but remained high even for longer sequences. Indeed, for simulated sequences, with 500 simulated neurons, from the estimated deformation of animal 1 (resp. 2 and 6), the accuracy decreased from 98.6 %* $\pm$ *0.3% (resp. 91.8 %* $\pm$ *0.3% and 97.8 %* $\pm$ *0.3%) for simulated sequences with 250 frames, to 87.5 %* $\pm$ *0.8% (resp. 85.5 %* $\pm$ *0.4% and 96.9 %* $\pm$ *0.3%) for 500 frames and 81.8 %* $\pm$ *1.1% (resp. 73.4 %* $\pm$ *1.1% and 92.0 %* $\pm$ *1.3%) for either 900 (animal 1) or 1000 (animal 2 and 6) frames. We also highlight that, in addition to high accuracy, the number of reconstructed tracks remained close to 500 even for longer sequence, with* $500.4\pm0.16$ *(250 frames),* $511.7\pm0.99$ *(500 frames) and* $515.2\pm0.98$ *(900 frames) reconstructed tracks for animal 1,* $511.5\pm1.25$ *(250 frames),* $518.9\pm1.06$ *(500 frames) and* $542.7\pm3.25$ *(1000 frames) reconstructed tracks for animal 2, and* $502.9\pm0.67$ *(250 frames),* $505.6\pm0.72$ *(500 frames) and* $515.7\pm2.3$ *(1000 frames) reconstructed tracks for animal 6.*

3- The expected output of the algorithm is not entirely clear. Fig1-6 seems to imply that one should expect a list of tracks spanning the entire length of the movie. However, from the Methods section, it seems possible that a short track can’t be concatenated with others. Is such a track eliminated entirely? Can the output contain partial tracks? In relation to this, since the elastic deformation is propagated forward only: is the number of expected tracks directly dependent on the number of cells detected at the first time step? Clarifications on this topic would make the technique more comprehensible and more directly applicable. Modifications of Fig 1-6 to show partial tracks would also clarify this point.

**Answer:** We have now added details in the methods Section about tracklet concatenation (see previous point 1) : Each final *long* track results from the concatenation of a given number of tracklets and do not necessarily span the entire time-lapse sequence. Also, some tracklets might not be concatenated to another one. In that latter case, the resulting final track would be composed by only one short tracklet. We agree that previous Figure 1 was confusing and we have now changed it to clearly show some partial final tracks:

***Figure 1****:* ***Multi-step EMC^2^*** ***for tracking single neuron activity in calcium imaging data. a-*** *Time-lapse imaging (N frames) of intermittent fluorescence activity of a neuron in a deforming environment (e.g. behaving animal).* ***b-*** *Fluorescent spots (neurons), that are significantly brighter than background, are automatically detected with a wavelet-based algorithm.* ***c-*** *Tracklets of detectable neurons are robustly reconstructed using probabilistic tracking algorithm (eMHT).* ***d-*** *Short tracklets of detectable particles are used to compute the elastic deformation of the field of view at each time frame. Associated detections in neuron tracklets are used as fiducials and the whole deformation is interpolated using a poly-harmonic thin-plate spline function. Forward- and backward-propagated positions of tracklet particle positions are shown with a thin blue line.* ***e-*** *After having corrected for the deformation of the field-of-view where neurons are embedded, gaps between the end- and starting-points of tracklets are closed by minimizing the global Euclidean distance between points (dotted line).* ***f-*** *Finally, complete single neuron tracks over the time-lapse sequence are obtained by applying the elastic transformation of the field-of-view to concatenated tracklets.*

**Minor revisions:**

1- I believe it would be worth mentioning in the title that the paper presents a new algorithmic technique for automated tracking. The focus on the Hydra test case could impair the visibility of the paper for some target communities.

**Answer:** We have now changed the title to: *“EMC^2^: A versatile algorithm for robust tracking of calcium dynamics from individual neurons in behaving animals”*

2- Similarly, I would suggest changing the keywords assigned to the paper to better reflect the fact that it presents a new tracking algorithm for intermittent signals and/or deforming media. This would help the dissemination of the technique to other fields.

**Answer:** We have now changed the keywords to: *“Single particle tracking; Deforming medium; Intermittent signal; Detectability; Spline; Calcium imaging; Neurons; Two-photon imaging, Hydra; Software “*

3- In several places, the authors mention the importance of calcium imaging in freely behaving animals. In the test case, the Hydras are still confined by a coverslip even though they do display deformations and normal behavior. Still, this should be mentioned clearly in the main text and care should be taken not to claim this example as a “freely behaving animal”.

**Answer:** We have now replaced “freely behaving” by “behaving” animal throughout the manuscript.

4- In Fig 1-5, the meaning of the arrows is not clear. I imagine they show the “decisions” that have to be made to concatenate short tracks but, as presented, these arrows seem mostly random.

**Answer:** We have now modified the Figure 1 and replaced arrows in Fig. 1e with a dotted black line showing the optimal concatenation of short tracklets. In Fig. 1d, we have also highlighted the forward- and backward-propagated positions of tracked particles with a thin blue line.

5- Between p8 and p9, the authors claim that “EMC2 contains only one free parameter” which is described as d_max but Methods also introduce gap_max as a second free parameter. Clarification is needed on this point.

**Answer:** EMC^2^ actually contains two free parameters, and we have now changed this sentence with: *“EMC^2^ contains only two free parameters: the maximal distance between forward propagated end-points and backward-propagated starting-points of short tracklets for concatenation, and, to avoid important error propagation, a maximum time-lag between ending- and starting-point candidates.”*

6- As described above, information on the number of obtained tracks is given for the tracking of Hydra neurons but not for the synthetic data. This should be added either in Fig3 or in the corresponding text.

**Answer:** In addition to the accuracy percentage of reconstructed tracks with EMC^2^, we have now added the number of simulated and reconstructed tracks throughout the manuscript. The number of tracks reconstructed with EMC^2^ is always very close to the number of simulated tracks.

7- In Fig4, a figure showing at least one example of silhouette scores versus number of clusters would be nice to support the claim that “We found 2 or 3 neuronal ensembles in each movie”.

**Answer:** We have added a figure showing one example of silhouette scores in Figure 4.

8- In this section and at the end of the discussion, the authors claim that the neuronal ensembles are “non-overlapping”. Although this was previously demonstrated, this claim is not supported by data in this paper. Precision on the fact that no, or very few, neurons were found to belong to two different ensembles (i.e. firing in more than 30% of ensemble spikes) would suffice to support this claim.

**Answer:** To show that extracted ensembles in Hydra are non-overlapping, we have measured, for each pair of ensembles and in each animal, the ratio between the number of neurons that were belonging to both ensembles, and the total number of neurons in the two ensembles. We obtained very low overlap ratio ($2.1\%\pm0.7 \%$, $2.4\%\pm0.4 \%$ and $1.6\%\pm0.6 \%$ respectively for CB-RP1, CB-RP2 and RP1-RP2 ensembles), confirming the non-overlap of the main neuronal ensembles in Hydra. We have added the following paragraph in section 4-b of the Results section:

*“Finally, we measured the overlap between ensembles, i.e. the proportion of single neurons that belonged to more than one ensemble. For each pair of ensembles, we computed the ratio between the number of shared neurons and the total number of neurons in both ensembles. We obtained ratio equal to* $2.1\%\pm0.7 \%$*,* $2.4\%\pm0.4 \%$ *and* $1.6\%\pm0.6 \%$ *respectively for CB-RP1, CB-RP2 and RP1-RP2 ensemble overlap. These very low values confirm the non-overlap of main neuronal ensembles in Hydra [14].”*

9- In fig4-f, it seems that the three ensembles come from the same experiment (3rd line in Table3) but the three pictures are different. Wouldn’t the case be made even stronger if the three ensembles were labeled in a single image?

**Answer:** The three ensembles are almost never active at the same time, this is why we chose three different times (and animal poses) to show these three ensembles *in action*.

10- Figure subpanels labelling should be added to Fig3 and homogenized between figures.

**Answer:** We have homogenized figure labeling.

11- In the last paragraph of the discussion section, the authors claim that “EMC2 is a robust and versatile tracking algorithm”. This claim of versatility needs to be further supported by discussion or demonstration of its applicability to different situations.

**Answer:** As stated in previous Major comment 1, we have now highlighted the versatility of our tracking method by:

i. Changing the title
ii. Adding a paragraph in the Introduction section to clearly state that our tracking method is in line with several technological developments to robustly track neurons in many model organisms
iii. Using the EMC^2^ method to track single neurons in two-photon imaging of mouse visual cortex.
iv. Discussing the possible extension of EMC^2^ to three-dimensional imaging of other animal models.

12- In Material and Methods, section 1, the explanation of the assignment problem that concatenates tracks is not very detailed. In particular, the constraints applied to i and j are not clear. What if a track has no other track within the d_max distance? What if concatenation doesn’t lead to full tracks?

**Answer:** We have now added details about tracklet concatenation in the Material and Methods (see previous points): if a tracklet is at distance > d_max of any other tracklet, it is not associated to any other tracklet and therefore, the associated final track will be composed by only one tracklet.

13- I have never used Icy before but tried to apply EMC2 to my own data on this platform. The code works but using it is not straightforward especially for people not used to programing. Time allowing, a more detailed, step-by-step protocol would most likely greatly increase the dissemination of the technique.

**Answer:** We thank referee for having pointed-out that EMC^2^ could be more user-friendly. We have added a step-by-step tutorial in the supplemental material of the paper. Also, to reach a broader panel of potential users, we are actively working on a Python version of the EMC^2^ pipeline

**Reviewer #2:**

Lagache and colleagues present a complete user-friendly software package for tracking neurons during short calcium recordings in moving and deforming hydra. They demonstrate that their method can track neurons even when those neurons are inactive, without the need for an additional fluorescent marker. The development of effective algorithms that are integrated into user-friendly and open source software packages like Icy is very valuable for the community.

There are two substantive concerns.

1) It is unclear how the method performs on recordings longer than 25 seconds.
I could not find any description of the effective recording length for the validation performed against synthetic data with empirically derived noise, so I don't know if that was greater than 25s. But it is important to show that the method can work over longer recordings because calcium recordings are often many minutes long, and the sequential nature of this method would seem to be particularly susceptible to accumulating errors over time. For example, I might reasonably expect to accumulate errors in stitching tracks together such that after a few minutes a significant fraction of neurons may now be assigned different labels at the end of the recording compared to the beginning. One way the authors could address this concern is by showing how performance degrades over recording length

when validated against realistic synthetic data.

**Answer:** To validate the robustness of EMC^2^ for longer movies, but also for other parameters such as the percentage of stable (i.e. non-blinking particles) or the number (density) of particles, we have measured the sensitivity of EMC^2^ to these three parameters (new Figure 3-d,e&f, reproduced below). Overall, these simulations show that EMC^2^ is robust to the percentage of stable particles, and the length of the sequence, but rapidly deteriorates as the number of particles (actually their density) decreases. Indeed, in simulations with very few simulated particles, the forward and backward deformation of the field-of-view cannot be accurately computed due to the lack of fiducial points, leading to important error propagation and erroneous tracklet concatenation.

*“Figure 3:* ***Testing EMC^2^ robustness with synthetic simulations.****…(d-e) Using “Hydra-like” synthetic deformation, we measured the accuracy of EMC2 for increasing proportion of stable (i.e. non-blinking) particles (neuronal cells) and increasing number of simulated particles. (f) After having estimated the deformation-field in three different animals (animal 1 (red), 2 (orange) and 6 (green)), we measured the accuracy of EMC2 for simulated sequences with increasing length (250, 500 and 900 or 1000 frames).”*

We have also added the following paragraphs in the Result section 3.b “Synthetic time-lapse sequences” of the manuscript:

*First, we found that even without stable cells (*$\alpha_{stable}=0$*), the accuracy of EMC^2^ remained high (77.7 %* $\pm$ *3.6% (398.8* $\pm$ *18.4 correct tracks over 513.3* $\pm$ *1.7 reconstructed tracks), and rapidly increased to 91.8 %* $\pm$ *1.2% for* $\alpha_{stable}=5\%$*, before reaching a plateau above 95% accuracy for* $\alpha_{stable}\geq10\%$*. Conversely, we found that EMC^2^ was very sensitive to the number of neurons, with poor performance for very few neurons (for simulations with only 10 neurons, the accuracy was 11.6 %* $\pm$ *0.6% (5.6* $\pm$ *0.3 correct tracks over 49.8* $\pm$ *0.8 reconstructed tracks)). The accuracy rapidly increased to > 90% when more than 100 neurons were simulated. Tracking errors in simulations with few neurons are due to the inaccurate estimation of the deformation field, and iterative error propagation, when few fiducial source and target points are used. Finally, we measured how the performance of the EMC^2^ decreased with the length of the simulated sequence, for three different animals (animal 1, 2 and 6 see Table 3). We chose animals with highest number of neurons so that we could accurately estimate, and therefore simulate, their body deformation. We found that the EMC^2^ accuracy slowly decreased with the sequence length but remained high even for longer sequences. Indeed, for simulated sequences, with 500 simulated neurons, from the estimated deformation of animal 1 (resp. 2 and 6), the accuracy decreased from 98.6 %* $\pm$ *0.3% (resp. 91.8 %* $\pm$ *0.3% and 97.8 %* $\pm$ *0.3%) for simulated sequences with 250 frames, to 87.5 %* $\pm$ *0.8% (resp. 85.5 %* $\pm$ *0.4% and 96.9 %* $\pm$ *0.3%) for 500 frames and 81.8 %* $\pm$ *1.1% (resp. 73.4 %* $\pm$ *1.1% and 92.0 %* $\pm$ *1.3%) for either 900 (animal 1) or 1000 (animal 2 and 6) frames. We also highlight that, in addition to high accuracy, the number of reconstructed tracks remained close to 500 even for longer sequence, with* $500.4\pm0.16$ *(250 frames),* $511.7\pm0.99$ *(500 frames) and* $515.2\pm0.98$ *(900 frames) reconstructed tracks for animal 1,* $511.5\pm1.25$ *(250 frames),* $518.9\pm1.06$ *(500 frames) and* $542.7\pm3.25$ *(1000 frames) reconstructed tracks for animal 2, and* $502.9\pm0.67$ *(250 frames),* $505.6\pm0.72$ *(500 frames) and* $515.7\pm2.3$ *(1000 frames) reconstructed tracks for animal 6.*

Hydra is particularly challenging as it experiences major and rapid deformation. Therefore, the accuracy of EMC^2^ might not be sufficient for Hydra time-lapse sequences with many more than few thousands of frames. To track cells over longer periods of time, animal immobilization, or at least confinement, should be needed [19]. In other experiments where neurons are less motile, such as in (head)-fixed animals, tracking is easier and neuronal activity could be robustly monitored over much longer time. To show the potential application of EMC^2^ to other, simpler, calcium imaging datasets, we tracked single neurons in targeted cortical regions of mice with two-photon microscopy. Even if the head of the animal is fixed under the two-photon microscope, the residual motion of neurons requires image post-processing (image registration and neuronal cell mask segmentation typically [10, 12]) to accurately measure single cell activity. We show here that EMC^2^ is an automatic, fast and robust tracking-based alternative to traditional image processing. Our results are summarized in the Results section 4-a “*Monitoring neuron activity with two-photon microscopy in mouse cortex*” and new Figure 4 (reproduced hereafter). In two-photon imaging, neuron trajectories can be modeled with stochastic confined motion, and are much easier to track than neurons in deforming animals like *C. Elegans* or *Hydra*. Therefore, the validation of EMC^2^ in these two extreme scenarios show that our method is sufficiently versatile to be applied in most, if not all, animal models.

***Figure 4: Monitoring calcium activity from individual neurons in mouse visual cortex with two-photon imaging and EMC^2^.*** *a- Two-photon imaging of calcium activity from individual neurons in visual cortex of awake mice is performed at Day 1, Day 2 and Day 46 during 5 minutes at 12.3 Hz. Tracking of neuron positions is performed with EMC^2^ and reveals an important turn-over of active neurons across days. Examples of neurons that are active at Day 1, Day 2 or Day 46 are respectively highlighted with red, green or blue arrows. Neurons active at Day 1&2 are highlighted with yellow arrows, at Day 2&46 with cyan arrows, and at Day 1,2&46 with white arrows. The median number of active neurons each day is also plotted (n=4 animals). The number of neurons that are active from Day 1, 2 or 3 are respectively represented in red, green or blue. b- Single neuron trajectories can be modeled with confined stochastic motion. Two example trajectories are shown (green & blue trajectories) with a maximum excursion distance of ~ 4 pixels. Boxplots of single neuron displacement between two consecutive frames, and maximum excursion distance (in pixels) are plotted (n=590 trajectories).*

2) Authors should clarify novelty and correct description of a previous method.
A key selling point of the method is that it uses deformation defined by tracked neurons to infer the location of missing neurons during gaps. The text highlights this method extensively, as if it were novel. But method [9], Ngueyn et al, explicitly performs the same step. Like this method, [9] uses thin plate splines of tracked neurons to infer the position of missing neurons, see main text accompanying Fig 5 in that reference [9].

The authors make a special point to explicitly compare to reference [9] in detail (for example on p. 7) and they also compare to many other methods in Table 1 and throughout the manuscript, all of which is commendable. However, they incorrectly claim that [9] is " designed to link two sets of particle detections in consecutive time frames." But this is incorrect. The method in [9] is time independent, in the sense that all the recorded volumes could be shuffled in time without changing tracking performance. Similarly, Table 1 incorrectly states that ref [9] is not robust to long gaps. On the contrary, because it is time independent, ref [9] is robust to exceptionally long gaps.
The authors should clarify novelty and correct descriptions of the previous method.

**Answer:** We have, indeed, claimed incorrectly that the NeRVE method proposed in [1] was “designed to link two sets of particle detections in consecutive time frames” and we have therefore removed this statement in the manuscript. We have also modified the *cons* of NeRVE method in table 1 with “*High computational load. Not robust to many missing detections and long gaps in highly deforming environments*.”

The NeRVE method is based on point-set registration of neuron positions and robust hierarchical clustering to identify single neurons in the different time frames. The method can therefore close long tracking gaps but, as we showed in [2], it is not is not sufficiently robust for tracking neurons with intermittent calcium imaging in highly deforming animals like *Hydra*. When using the NeRVE method in *C. Elegans*, authors labeled neurons with calcium-insensitive probes and performed image pre-processing (animal “straightening” and mapping of neuron positions along the worm principal axis) was performed to reduce the effective deformation of the animal and ensure a sufficient accuracy of the tracking. To clarify the novelty of our approach, we have added the following paragraph in Results section (2- *EMC^2^ algorithm*):

*“We highlight that the Neuron Registration Vector Encoding (NeRVE) method developed to map single neurons in C. elegans [1] is not sufficiently robust for tracking neurons in calcium imaging of behaving Hydra as showed in [2]. The poor tracking accuracy of NerVE method when applied to Hydra is due to two main reasons. The first reason is that, contrary to C. elegans worm, one cannot map the Hydra neuron positions in cylindrical coordinates along the principal axis of the animal. This drastically reduces the effective deformation of C. elegans and thus, the complexity of neuron registration. The second main reason is the intermittent detectability of neurons in calcium imaging, compared to the calcium-insensitive RFP labeling used in [1]. Indeed, calcium imaging is related to neuron activity and leads to tracking gaps with longer and more variable lengths. Altogether, the higher animal deformation and the stochastic detectability of neurons result in an important error-propagation during neuron registration. This can explain the low accuracy of the global vector clustering method of the NeRVE algorithm when applied to Hydra [2]. To increase the robustness and accuracy of single neuron tracking, we rather implement, in EMC^2^, a local concatenation of short tracks (tracklets) after having propagated forward and backward the estimated deformation of the field of view. “*

Additional technical concern:

There seems to be an inconsistency with Figure 4. It shows calcium activity lasting 1,400 s or 23 mins, whereas the main text and Table 3 suggests that recordings lasted less than 2 mins (1000 frames @ 10hz). Please resolve this inconsistency.

**Answer:** We have corrected this typo and changed 1,400 s to 1,400 frames (140 s) in that Figure.

References

1.	Nguyen, J.P., et al., *Automatically tracking neurons in a moving and deforming brain.* PLoS Comput Biol, 2017. **13**(5): p. e1005517.

2.	Lagache, T., et al., *Tracking Activity In A Deformable Nervous System With Motion Correction And Point-Set Registration.* bioRxiv, 2018: p. 373035.

3.	Ahrens, M.B., et al., *Whole-brain functional imaging at cellular resolution using light-sheet microscopy.* Nature methods, 2013. **10**(5): p. 413-420.

4.	Seelig, J.D., et al., *Two-photon calcium imaging from head-fixed Drosophila during optomotor walking behavior.* Nature methods, 2010. **7**(7): p. 535-540.

5.	Yang, W. and R. Yuste, *In vivo imaging of neural activity.* Nat Methods, 2017. **14**(4): p. 349-359.

6.	Kim, D.H., et al., *Pan-neuronal calcium imaging with cellular resolution in freely swimming zebrafish.* Nature methods, 2017. **14**(11): p. 1107-1114.

7.	Nguyen, J.P., et al., *Whole-brain calcium imaging with cellular resolution in freely behaving Caenorhabditis elegans.* Proc Natl Acad Sci U S A, 2016. **113**(8): p. E1074-81.

8.	Bouchard, M.B., et al., *Swept confocally-aligned planar excitation (SCAPE) microscopy for high-speed volumetric imaging of behaving organisms.* Nature photonics, 2015. **9**(2): p. 113-119.

9.	Thevenaz, P., U.E. Ruttimann, and M. Unser, *A pyramid approach to subpixel registration based on intensity.* IEEE transactions on image processing, 1998. **7**(1): p. 27-41.

10.	Dubbs, A., J. Guevara, and R. Yuste, *moco: Fast motion correction for calcium imaging.* Frontiers in neuroinformatics, 2016. **10**: p. 6.

11.	Yu, X., et al., *Fast deep learning correspondence for neuron tracking and identification in C. elegans using synthetic training.* arXiv preprint arXiv:2101.08211, 2021.

12.	Pnevmatikakis, E.A., et al., *Simultaneous Denoising, Deconvolution, and Demixing of Calcium Imaging Data.* Neuron, 2016. **89**(2): p. 285-99.

13.	Nejatbakhsh, A., et al. *Demixing Calcium Imaging Data in C. elegans via Deformable Non-negative Matrix Factorization*. in *International Conference on Medical Image Computing and Computer-Assisted Intervention*. 2020. Springer.

14.	Dupre, C. and R. Yuste, *Non-overlapping Neural Networks in Hydra vulgaris.* Curr Biol, 2017. **27**(8): p. 1085-1097.

15.	Vaadia, R.D., et al., *Characterization of proprioceptive system dynamics in behaving Drosophila larvae using high-speed volumetric microscopy.* Current Biology, 2019.

16.	Beatson, R., M.J.D. Powell, and A. Tan, *Fast evaluation of polyharmonic splines in three dimensions.* IMA Journal of Numerical Analysis, 2007. **27**(3): p. 427-450.

17.	Kuhn, H.W., *The Hungarian method for the assignment problem.* Naval research logistics quarterly, 1955. **2**(1‐2): p. 83-97.

18.	Jonker, R. and T. Volgenant, *Improving the Hungarian assignment algorithm.* Operations Research Letters, 1986. **5**(4): p. 171-175.

19.	Badhiwala, K.N., et al., *Microfluidics for electrophysiology, imaging, and behavioral analysis of Hydra.* Lab on a Chip, 2018. **18**(17): p. 2523-2539.
