# Supplementary material for: Tracking calcium dynamics from individual neurons in behaving animals
Source: PLoS Comput Biol. 2021 Oct 8;17(10):e1009432. doi: 10.1371/journal.pcbi.1009432 (PMC8528277; doi:10.1371/journal.pcbi.1009432)
Supplement: Attachment — Submitted filename: Cover_Letter_Revision_2.docx [file pcbi.1009432.s005.docx]

Thursday, April 22^nd^, 2021

Dear Professor Gutkin,

We are submitting our revised manuscript entitled " *EMC^2^: A versatile algorithm for robust tracking of calcium dynamics from individual neurons in behaving animals*" for your consideration as an Article in PLOS Computational Biology. We would like to thank you for giving us the opportunity to respond to the remaining concerns of reviewer 2 about the sustained performance of our tracking algorithm for longer sequences.

To answer his/her concerns we have now run simulations for synthetic sequences with 4 minutes length (2400 frames). New simulations demonstrate that the accuracy of EMC^2^ remains high (i.e. > 80%) for up to 2 to 4 minutes depending on the animal deformation used for simulations. While this performance is sufficient for the analysis performed in section 4-b “Characterizing neuronal ensembles in behaving *Hydra”* of the manuscript, we acknowledged in the manuscript that further hardware implementation such as dual-color imaging of neurons with calcium-insensitive dyes, or partial immobilization of the animal would be required for the accurate tracking of neurons over longer time (several minutes or hours). Nevertheless, the possibility for future improvement should not diminish the merit of the current algorithm and pipeline, which greatly supersedes existing approaches. Indeed, we would like to emphasize that intermittent calcium imaging in highly deforming Hydra is presumably one of the worst-case scenario for tracking purposes. Therefore, tracking neurons over minutes with reasonable accuracy is already a technical feat that paves the way to longer and more robust monitoring of the calcium activity of individual neurons in most, if not all, animals.

Looking forward to a favorable response

Yours sincerely,

Thibault Lagache and Rafael Yuste

**Reviewer #2:**

In this revision Lagache and colleagues have made important revisions and clarifications. It remains unclear, however, whether the method can track neurons in hydra over a sufficiently long timescale to be useful. The authors specifically motivate their work by the need to "achieve robust and automatic tracking of individual cells over long time-lapse sequences." Most calcium activity experiments are for at least a few minutes, and recent published work by this group recorded calcium activity in moving hydra for 2 hours (Yamamoto and Yuste 2020, see Fig 3). It is therefore imperative that the authors show what accuracy to expect on the 5 min timescale and beyond.
With synthetic data, the authors now show that their method's accuracy drops from 97% for 250 frames (presumably 25s) to an accuracy of ~80% for 1000 frames (presumably 100s). It seems possible that accuracy will continue to drop dramatically as the timing approaches 5 mins.

Related: synthetic data is reported in terms of frames, but the authors should instead report the equivalent time in seconds, based on the timescale of the motion in the synthetic data. The authors should make this easy to find in the text and figure or caption. If the method is unable to achieve reasonable accuracy on the multiple minute timescale, the authors should scale back their claims, and it may also reduce the significance of the work.

**Answer:**

We would like to highlight that Hydra data, with its tremendous deformations, can be considered the worse possible scenario for neuronal tracking. Just to clarify: all published Hydra papers have performed tracking by hand of a few hundred frames, or, like in the Yamamoto paper mentioned, merely measuring the fluorescence intensity for the entire body, without any neuronal tracking. Thus, to show any robust automatic tracking of Hydra data is already a major advance.

Nevertheless, in response to the reviewer, we have now run simulations for longer synthetic sequences (up to 4 minutes length (2,400 frames at 10 Hz)). New simulations (Figure 3-f see hereafter, and new Table 3) demonstrate that the accuracy of EMC2 remains high (i.e. > 80%) for up to 2 to 4 minutes depending on the animal deformation used for simulations. For longer simulations, the accuracy dropped below 80% but remained above $\sim70\%$ for all animals and the entire length of the simulation (4 minutes). While this performance of EMC^2^ is sufficient for the analysis performed in section 4-b “Characterizing neuronal ensembles in behaving *Hydra”* of the manuscript, where the length of analyzed movies was of ~100 s (1,000 frames), we have now acknowledged, in the manuscript, that further hardware implementation such as dual-color imaging of neurons with calcium-insensitive dyes, or partial immobilization of the animal would be required for the accurate tracking of neurons over several minutes or hours. Nevertheless, the possibility for future improvement should not diminish the merit of the current algorithm and pipeline, which greatly supersedes existing approaches.

We have added the following paragraph at the end of Results section 3 in the manuscript:

*“We found that the EMC^2^ accuracy slowly decreased with the sequence length but remained high (i.e. >80%) for up to 2 to 4 minutes of simulation at 10 Hz, depending on the animal (animal 1 ~ 1200 frames = 2 min., animal 4 ~ 1500 frames = 2 min. 30s and animal 6 ~ 2400 frames = 4 min.). For longer simulations, the accuracy dropped below 80% but remained above* $\sim70\%$ *for all animals and the entire length of the simulation (4 minutes). In addition to preserved accuracy, the number of reconstructed tracks remained close to 500 even for longer sequences (Fig. 3.f and Table 3). Therefore, EMC^2^ tracking algorithm allows the robust, automatic tracking of individual neurons over few minutes in highly deforming Hydra model. For tracking neurons over longer time (several minutes or hours), hardware implementations such as dual-color imaging with calcium-insensitive dye or partial immobilization of the animal would be required.”*

Given that Hydra data is likely the worse possible scenario, we hope that the reviewer agrees that tracking Hydra neurons over minutes with reasonable accuracy is already a technical feat that paves the way to longer and more robust monitoring of the calcium activity of individual neurons in most, if not all, animals.

***Figure 3: Testing EMC^2^ robustness with synthetic simulations.*** *For each simulated type of motion (confined diffusion (****a****), linear motion (****b****) and “Hydra-like” elastic deformation (****c****)), we simulated the stochastic firing of neuronal ensembles and corresponding fluorescence dynamics in synthetic time-lapse sequences (see Materials and Methods for details). We then compared the performances of EMC^2^ (blue) with TrackMate (no motion correction (red) or linear motion correction (magenta)). P-values are obtained with the Wilcoxon rank sum test over n=10 simulations in each case. (****d-e****) Using “Hydra-like” synthetic deformation, we measured the accuracy of EMC^2^ for increasing proportion of stable (i.e. non-blinking) particles (neuronal cells) and increasing number of simulated particles. (****f****) After having estimated the deformation-field in three different animals (animal 1 (black), 4 (blue) and 6 (green)), we measured the accuracy of EMC^2^ for simulated sequences with increasing length (25, 50, 100 and 240 seconds. Imaging and simulations were performed at 10 Hz). For comparison purposes, the performance of TrackMate algorithm for 25 seconds (animal 1), extracted from* ***(c),*** *is shown.*

**Table 3: Results of synthetic simulations (Hydra-like deformation, increasing length)**

|  | **250 frames (25 s.)** | | **500 frames (50 s.)** | | **1000 frames (100 s.)** | | **2400 frames (240 s.)** | |
| --- | --- | --- | --- | --- | --- | --- | --- | --- |
|  | *Accuracy* | *Nb. Tracks* | *Accuracy* | *Nb. Tracks* | *Accuracy* | *Nb. Tracks* | *Accuracy* | *Nb. Tracks* |
| **Animal 1** | $98.6\pm0.3\%$ | $500.4\pm0.2$ | $87.5\pm0.8\%$ | $511.7\pm1.0$ | $83.8\pm1.8\%$ | $512.0\pm3.1$ | $67.4\pm4.1\%$ | $538.8\pm8.0$ |
| **Animal 4** | $98.0\pm0.2\%$ | $503.5\pm0.4$ | $95.9\pm0.4\%$ | $507.9\pm1.1$ | $88.7\pm1.2\%$ | $521.5\pm2.6$ | $66.7\pm2.5\%$ | $571.9\pm8.5$ |
| **Animal 6** | $97.8\pm0.2\%$ | $502.9\pm0.7$ | $96.9\pm0.3\%$ | $505.6\pm0.7$ | $92.0\pm1.3\%$ | $515.7\pm2.3$ | $81.7\pm1.9\%$ | $540.7\pm5.4$ |

References

1.	Lagache, T., et al., *Tracking Activity In a Deformable Nervous System With Motion Correction and Point-Set Registration.* bioRxiv, 2018: p. 373035.

2.	Nguyen, J.P., et al., *Automatically tracking neurons in a moving and deforming brain.* PLoS Comput Biol, 2017. **13**(5): p. e1005517.
