# Supplementary material for: Tracking calcium dynamics from individual neurons in behaving animals
Source: PLoS Comput Biol. 2021 Oct 8;17(10):e1009432. doi: 10.1371/journal.pcbi.1009432 (PMC8528277; doi:10.1371/journal.pcbi.1009432)
Supplement: Attachment — Submitted filename: Cover_Letter_Revision_3.doc [file pcbi.1009432.s006.doc]

Thursday, June 24^th^, 2021

Dear Professor Gutkin,

We are submitting our revised manuscript entitled " *EMC^2^: A versatile algorithm for robust tracking of calcium dynamics from individual neurons in behaving animals*" for your consideration as an Article in PLOS Computational Biology. We would like to thank you for giving us the opportunity to respond to the remaining concerns of reviewer 2 about the performance of our tracking algorithm for long sequences, contrasting it to the methods developed for *C. elegans* [1-5]. These latter methods are based on the statistical mapping of neurons to a fixed reference set of positions in the animal, and are therefore *time-independent* (i.e. their performance is not supposed to decrease with time) as images are registered independently from each other to a reference set of positions extracted from reference frames, in the time-lapse sequence or online cell atlases [4, 5]. Therefore, they require the stability of the positions of cells in the animal(s).

*C.elegans*, with its fixed structure with stereotyped number of cells and positions, is an exception in the animal world. In *Hydra*, like in most animals, the number and position of neurons differ from one animal to another [6]. In addition, the definition of reference cell positions within a single animal (i.e. the mapping of all neuron positions within reference frames) is essentially impossible due to **a-** the major and incessant deformations of the animal (the length of the animal is reduced by more than half during longitudinal contraction!), **b-** the 2D imaging of transparent 3D tissues that induces apparent changes in neuron positions even between comparable poses of the animals and **c-** the intermittent detectability of neurons in calcium imaging. For all these reasons, *time-independent* mapping methods cannot be applied to *Hydra*, as we showed in [7].

Because of this, we used the more versatile single-particle-tracking (SPT) framework to develop EMC^2^. SPT is based on the sequential linking cell detections along time [8]. As pointed out by reviewer 2, a drawback of SPT methods is their decreasing accuracy in longer time-lapse sequences, as the probability of association error between detections and existing tracks increases. That said, we emphasize that EMC^2^ performances supercede the state-of-the art SPT methods and that, as it stands, EMC^2^ made possible the tracking of individual neurons over few (~2-3) minutes in Hydra, which is probably the worst-case scenario for tracking purposes. This allowed us to statistically characterize, for the first time, the main three neuronal ensembles of the animal. Tracking neurons over minutes in *Hydra* with reasonable accuracy is already a technical feat that paves the way to the robust monitoring of single neuron activity over minutes. Finally, the important versatility of EMC^2^ allows the tracking of neurons in any other animal model, and also, as underlined by the first referee, the tracking of objects in many other bioimaging problems such as moving cells in remodeling tissues.

To answer the remaining concerns of Reviewer 2, we have now:

-	softened claims by removing terms like "long time lapse sequences", "long-term" monitoring or "long" gaps throughout the manuscript.

-	added a paragraph “*Statistical Mapping of Neuron Positions versus Single-Particle-Tracking*” in the Results section to clearly describe the “*time-independent*” mapping methods developed for *C. Elegans*. In this paragraph, we also explained why mapping methods cannot be directly applied to *Hydra*, justifying the need for a more versatile single-particle-tracking approach, even though SPT performance decreases in time due to erroneous associations of detected particles to existing tracks.

-	added methods related to the statistical mapping of neurons’ positions in *C. Elegans* [4, 5] in Table 1. We have also indicated the used framework (Mapping or SPT) for each tracking method, and specified that mapping methods are “*time-independent (i.e. robust even in long time-lapse sequences)*”

-	discussed potential future implementations that could help to bridge beyond the minutes’ timescale. These implementations include hardware solutions, such as dual-color imaging with calcium-insensitive dye or partial immobilization of the animal [9], and software solutions, such as the use of stereotypical poses of the animal [10] as reference frames for mapping the (almost) stable positions of neuronal sub-sets.

A point-by-point answer to reviewer’s concerns is attached.

Looking forward to a favorable response

Yours sincerely,

Thibault Lagache and Rafael Yuste

**Reviewer #2:**

With further simulation studies, the authors now show that their method's performance degrades as recording duration increases. For Hydra, the method is only suitable for very short recordings (<5 min timescale). This contradicts claims made in the abstract and throughout the text related to "long time lapse sequences" or "long-term" monitoring. Claims about closing potentially "long" gaps also lack evidence. In short there is a disconnect between many of the authors' claims and the evidence.

Evidence does not support the authors claim that that this method "greatly supersedes existing approaches." In contrast, other methods (NeRVE from Ngueyn et al. 2017; Conditional Random Fields from Chaudhary et al. elife 2021) are fundamentally time-independent and thus maintain the same performance regardless of recording duration. The authors need to revise their comparisons to other methods with a frank assessment of this limitation, which seems to arise from the sequential time-dependent nature of the algorithm. For example, it would be relevant to report in Table 1 whether each algorithm is time-dependent or not.

Crucially, the authors need to better justify why this method is meritorious despite being limited to only very short recordings. To be compelling the authors should provide specific use cases, and also include a discussion of why and how errors accumulate with time and what fundamentally about the algorithm imposes this limitation.

**Answer**: The referee has raised a good point: the framework used for tracking neurons in *C. Elegans* [1-5] is, indeed, very different from the one we used in EMC^2^ for tracking neurons in *Hydra*. Most methods that have been developed for *C. Elegans* are based on the statistical mapping of neurons to a reference set of positions in the animal. Therefore, these methods are “*time-independent*” as the different images of the time-lapse sequence are registered independently from each other to a reference set of positions. In these methods, reference positions are extracted from reference frames, and cell identities can be either obtained from recent stereotyped fluorescent color map to identify all neurons in *C. Elegans* (NeuroPAL [11]) or online cell atlases (WormAtlas [12] and OpenWorm [13]). Therefore, all the developed methods heavily rely on the stable repertoire of neurons (type and position) **within single worms**, when mapping is used for tracking neurons along time [1-3], and **across different worms** when different animals or strains are compared [4, 5].

Unfortunately, in *Hydra*, like in most animals, the number and position of neurons **differ from one animal to another** [6], and there is no such tools as cell atlases or multi-color animal strains to map the generic positions of, at least, some neuron types. Moreover, the definition of refence cell positions **within a single animal** (i.e. the mapping of all neuron positions within reference frames) is prevented from **a-** the important and incessant deformations of the animal (typically, the length of the animal is reduced by more than half during longitudinal contraction), **b-** the 2D imaging of transparent 3D tissues that induces apparent changes in neuron positions even between two similar poses of the animal.

Also, the mapping methods developed for *C. Elegans* can accommodate slight changes in neuron positions due to animal deformation (position noise), and neuron numbers due to missing or spurious detections (counting noise). But the important Hydra deformation and, above all, the intermittent and sparse detectability of neurons in calcium imaging, hinder the applicability of *C. Elegans* tracking methods. For example, the NeRVE method does not account for the neurons that are detected in less than 40% of the frames of the time-lapse sequence [1], which is the case of most tracked neurons in *Hydra*. Another example is given by the maximum position and count noise that are used for testing the performances of fDLC mapping method in [5]: “*spurious neurons are randomly added, and some true neurons are randomly removed, for up to 20% of the observed neurons. To introduce variability associated with animal pose, we randomly deform the centerline of the head. Lastly, to account for variability in animals’ size and orientation, a random affine transformation in XY plane is applied that rescaled the animal’s size by up to 5%.”,* which are much smaller than the position and counting noises encountered in *Hydra* imaging.

For all the aforementioned reasons, these mapping methods cannot be applied to Hydra (as we showed in [7]). Therefore, we used the more versatile single-particle-tracking (SPT) framework to develop EMC^2^. SPT methods sequentially link cell detections along time [8] and, as pointed out by referee 2, a drawback of SPT is that their performance decreases with the sequence length, as the probability of association error between detections increases.

That said, we would like to emphasize that

- EMC^2^ performances largely supersede the state-of-the art SPT methods (Bayesian eMHT [14], *TrackMate* [15]…) thanks to iterative correction of animal deformation
- Tracking performances of EMC^2^ are comparable to those obtained with statistical mapping in *C. Elegans* :

***NeRVE [1]***

Data : 2 volumetric time-lapse imaging acquired at 3 Hz (i.e. 3 volumes per second) during 4 and 8 minutes respectively. Calcium-insensitive marker.

- Authors compared 70 manually annotated with 119 automatically detected neurons (i.e. significant clusters in NeRVE tracking). Among the 70 manually annotated neurons, 52 neurons were correctly tracked over the entire time-lapse sequence (Fig. 6B), the other 18 neurons were correctly tracked in more than 95% of the frames.

***Graphical model framework [4]***

Data (Figure 4) : n=35 time-lapse volumes of 16 cells imaged at 1 Hz over 1,000 frames (1000 s). GCaMP imaging (intermittent detectability).

- Accuracy of cell identification is measured using OpenWorm atlas. Accuracy was equal to 98 %, and dropped to 88% when 3 random cells are removed randomly from the dataset (to simulate count noise). These results obtained for a subset of neurons in almost immobile animals should be compared to the results obtained with EMC^2^ in two-photon microscopy of mouse cortex (98% accuracy over >50 cells and 5 min. recording at 12.3 Hz (~3,700 frames).

***fDLC [5]***

Data (figure 3): same dataset than in NeRVE (4 or 8 min. sequence acquired at 3Hz).

- The test protocol is *“201 different time points during this recording to form 200 pairs of configurations upon which to evaluate the fDLC model. Each pair consisted of a test and template. The template was always from the same time point t, while the test was taken to be any of the other 200 time points”*
- Accuracy was measured using manual annotation: *« Across the 200 pairs, the fDLC model showed an average accuracy of 80.0%”*
- As it stands, EMC^2^ already allowed us to robustly track hundreds of neurons over a few minutes in Hydra, which is probably the worst-case scenario for tracking purposes. We could then, for the first time, statistically characterize the main 3 neuronal ensembles of the animal. Moreover, the versatility of the SPT framework permits the tracking of individual neuron activity in most, if not any, other animal models, as we exemplified with two-photon imaging of mouse cortex. For this latter application, the accuracy of EMC^2^ was in line with other, more specialized tracking techniques such as Deformable Non-negative Matrix Factorization [2], or graphical model framework [4].

Finally, multiple hardware, such as two-color imaging with calcium insensitive dyes or partial immobilization of the animal, and software, such as the use of stereotypical poses of the animal to decrease the sensitivity of EMC^2^ to sequence length, could be potentially implemented in the near future to further improve the accuracy of EMC2 and help tracking neurons over several minutes.

**Manuscript changes:**

- We have now softened claims by removing terms like "long time lapse sequences", "long-term" monitoring or "long" gaps throughout the manuscript.
- We have now added a paragraph “*Statistical Mapping of Neuron Positions versus Single-Particle-Tracking*” in the Results section to clearly describe the “time-independent” mapping methods developed for *C. Elegans*. In this paragraph, we also explained why mapping methods cannot be directly applied to *Hydra*, justifying the need for a more versatile single-particle-tracking approach, even though SPT performance decreases in time due to erroneous associations of detected particles to existing tracks.

*1-	Statistical Mapping of Neuron Positions versus Single-Particle-Tracking*

*Most of the methods that have been developed for tracking single neuron activity in the well-documented animal model C. Elegans are based on the statistical mapping of neuron positions to a reference set of coordinates within the animal [10, 16, 22]. An important advantage of these methods is their robustness to the length of the analyzed time-lapse sequence, as the different images are registered independently from each other to the reference set of positions. In these methods, reference positions are extracted from reference frames and cell identities can be either obtained from stereotyped fluorescent color map of all neurons (NeuroPAL [23]) or online cell atlases (e.g. WormAtlas [24] and OpenWorm [25]). Therefore, mapping methods heavily rely on the stable repertoire of neurons (position and/or type) within single worms, when mapping is used for tracking neurons along time [10, 18, 26], and even across different worms when different animals or strains are compared [16, 22].*

*Unfortunately, in Hydra, the number and position of neurons differ from one animal to another [27]. Moreover, even within a single animal, the accurate mapping of a referent set of neuron positions is prevented from the important and continual deformations of the animal (typically, the length of the animal is reduced by more than half during longitudinal contraction), and the 2D imaging of transparent 3D tissues that induces apparent changes in neuron positions even between stereotypical poses of the animal. Finally, the aforementioned mapping methods used in C. Elegans can accommodate moderate changes in the total number of neurons between frames due to missing or spurious detections (counting noise), but the intermittent and sparse detectability of neurons in calcium imaging, definitely hinder the applicability of mapping methods to Hydra [7].*

*Therefore, to track neurons in Hydra, we used the SPT framework. SPT methods sequentially link cell detections along time [8], with no need for reference set of positions. They are therefore more versatile than statistical mapping, and can handle the large deformations of Hydra and the intermittent detectability of cells in calcium imaging.”*

- In Table 1 – “*A review of tracking methods in bio-imaging”*, we have added methods related to the statistical mapping of neurons’ positions in *C. Elegans* [4, 5], and we have indicated the used framework (Mapping or SPT) for each tracking method. We have also specified that mapping methods are “*Time-independent (i.e. robust even in long time-lapse sequences)”*
- To highlight that decreased performance with time is an inherent drawback of SPT-based methods, we have added sentences in the Results’ paragraphs

*“2- Limitations of standard SPT algorithms*

*…the erroneous association of one detection to a track, or the premature ending of a track due to missing detection(s), can lead to important error propagation as detections are sequentially associated to existing tracks.”*

And

“*4b – Synthetic time-lapse sequences*

*The decreased performance of EMC2 for longer time-lapse sequences is an expected drawback of SPT-based tracking algorithms. Indeed, the probability of false associations between newly detected particles and existing tracks increases with time.”*

- Finally, in the Discussion section, we have presented potential future implementations that could help to bridge beyond the minutes’ timescale.

*“For tracking neurons over longer time (several minutes or even hours), hardware implementations such as dual-color imaging with calcium-insensitive dye or partial immobilization of the animal [48] would be required. Another possible software strategy for increasing the robustness of EMC2 tracking over long time would be the characterization and use of stereotypical poses of the animal [8] as reference frames for mapping the (almost) stable positions of neuronal sub-sets.”*

Minor, but important: it is imprecise to claim that accuracy "remained above ~70% for all animals" when Table 3 reports accuracy of 66.7% and 67.4% for two of the three animals.

Answer: We have now reported the mean accuracy (72%) over the three animals.

References

1.	Nguyen JP, Linder AN, Plummer GS, Shaevitz JW, Leifer AM. Automatically tracking neurons in a moving and deforming brain. PLoS Comput Biol. 2017;13(5):e1005517. Epub 2017/05/18. doi: 10.1371/journal.pcbi.1005517. PubMed PMID: 28545068; PubMed Central PMCID: PMCPMC5436637.

2.	Nejatbakhsh A, Varol E, Yemini E, Venkatachalam V, Lin A, Samuel AD, et al., editors. Demixing Calcium Imaging Data in C. elegans via Deformable Non-negative Matrix Factorization. International Conference on Medical Image Computing and Computer-Assisted Intervention; 2020: Springer.

3.	Nejatbakhsh A, Varol E, Yemini E, Venkatachalam V, Lin A, Samuel AD, et al. Extracting neural signals from semi-immobilized animals with deformable non-negative matrix factorization. bioRxiv. 2020.

4.	Chaudhary S, Lee SA, Li Y, Patel DS, Lu H. Graphical-model framework for automated annotation of cell identities in dense cellular images. Elife. 2021;10:e60321.

5.	Yu X, Creamer MS, Randi F, Sharma AK, Linderman SW, Leifer AM. Fast deep learning correspondence for neuron tracking and identification in C. elegans using synthetic training. arXiv preprint arXiv:210108211. 2021.

6.	Dupre C, Yuste R. Non-overlapping neural networks in Hydra vulgaris. Current Biology. 2017;27(8):1085-97.

7.	Lagache T, Lansdell B, Tang J, Yuste R, Fairhall A. Tracking Activity In a Deformable Nervous System With Motion Correction and Point-Set Registration. bioRxiv. 2018:373035.

8.	Chenouard N, Smal I, De Chaumont F, Maška M, Sbalzarini IF, Gong Y, et al. Objective comparison of particle tracking methods. Nature methods. 2014;11(3):281-9.

9.	Badhiwala KN, Gonzales DL, Vercosa DG, Avants BW, Robinson JT. Microfluidics for electrophysiology, imaging, and behavioral analysis of Hydra. Lab on a Chip. 2018;18(17):2523-39.

10.	Han S, Taralova E, Dupre C, Yuste R. Comprehensive machine learning analysis of. Elife. 2018;7. Epub 2018/03/28. doi: 10.7554/eLife.32605. PubMed PMID: 29589829; PubMed Central PMCID: PMCPMC5922975.

11.	Yemini E, Lin A, Nejatbakhsh A, Varol E, Sun R, Mena GE, et al. NeuroPAL: a multicolor atlas for Whole-Brain neuronal identification in C. elegans. Cell. 2021;184(1):272-88. e11.

12.	Altun ZF, Chen B, Wang ZW, Hall DH. High resolution map of Caenorhabditis elegans gap junction proteins. Developmental dynamics: an official publication of the American Association of Anatomists. 2009;238(8):1936-50.

13.	Szigeti B, Gleeson P, Vella M, Khayrulin S, Palyanov A, Hokanson J, et al. OpenWorm: an open-science approach to modeling Caenorhabditis elegans. Frontiers in computational neuroscience. 2014;8:137.

14.	Chenouard N, Bloch I, Olivo-Marin J-C. Multiple hypothesis tracking for cluttered biological image sequences. IEEE transactions on pattern analysis and machine intelligence. 2013;35(11):2736-3750.

15.	Tinevez J-Y, Perry N, Schindelin J, Hoopes GM, Reynolds GD, Laplantine E, et al. TrackMate: An open and extensible platform for single-particle tracking. Methods. 2017;115:80-90.
