# Supplementary material for: Tracking calcium dynamics from individual neurons in behaving animals
Source: PLoS Comput Biol. 2021 Oct 8;17(10):e1009432. doi: 10.1371/journal.pcbi.1009432 (PMC8528277; doi:10.1371/journal.pcbi.1009432)
Supplement: Attachment — Submitted filename: Cover_Letter_Revision_4.doc [file pcbi.1009432.s007.doc]

Friday, July 16^th^, 2021

Dear Professor Gutkin,

We are submitting our revised manuscript entitled "*Tracking calcium dynamics from individual neurons in behaving animals*" for your consideration as an Article in PLOS Computational Biology. We would like to thank you for giving us the opportunity to respond to the remaining concerns of reviewer 2

A point-by-point answer to reviewer’s concerns is attached.

Looking forward to a favorable response

Yours sincerely,

Thibault Lagache and Rafael Yuste

**Reviewer #1**: I thank the authors for the clarifications of time-dependent and time-independent methods following Reviewer 2's comments. I feel these clarifications now clearly define both the advantages and limitations of the EMC2 method in comparison with previously developped solutions. By clearly discussing in which situations, such as Hydra neuronal tracking, EMC2 provides improvement from existing methods, the authors now clearly provide the reader with both a novel tracking method and rational arguments for using it, or not, depending on her/his own experimental data.

**Answer:**  We thank reviewer #1 for her/his positive feedbacks on our manuscript.

Reviewer #2: The authors have taken important steps to bring their claims more into alignment with the evidence they present, but some problematic language remains.

• The phrase “Long time lapse sequences” still appears on p. 4 This should be removed because it implies that the method will address the “long time lapse” case, when it does not.

**Answer:**  We removed “over sufficiently long time-lapse sequences” from this sentence.

• The placement of new language about “…premature ending of a track due to missing detection(s), can lead to important error propagation” on p. 8 is problematic. That text appears in subsection “2 - Limitations of Standard SPT Algorithms,” but the authors claim incorrectly that EMC^2 overcomes those limitations of standard SPT algorithms in subsection “3 – EMC^2 Algorithm”: “To overcome these limitations, we have developed EMC2…” The authors need to revise this framing so that they do not incorrectly claim that they have overcome the limitations on accumulation of errors over time.

**Answer:**  We replaced

“To overcome these limitations, we have developed EMC2, a method and software to track single particles with intermittent detectability in cluttered environments. It is particularly well-suited for tracking single neuron activity with calcium imaging in a behaving animal.”

with

“To increase the capability of SPT algorithms to track single particles with intermittent detectability in cluttered and deforming environments, we have developed EMC2. This multi-step algorithm and software is particularly well-suited for tracking single neuron activity with calcium imaging in a behaving animal.”

• The claim (p.16) that EMC^2 is robust “independent of the type and complexity of particle motion” is not supported by the data. Indeed if this were true it would be extraordinary. I suspect that the performance of EMC^2, like all time-dependent tracking algorithms before it, is dependent on the amount and type of motion per frame, and with enough motion per frame its performance would degrade precipitously. Please qualify this claim or provide evidence to support it.

**Answer:**  We replaced

“independent of the type and complexity of particle motion” with ”for different types of particle motions”.

Minor, but important:

• This statement on p. 7 incorrectly implies that the sparse detectability of neurons is uniquely problematic for Hydra and not C. elegans or other organisms, when in fact this depends only on whether the animal is expressing a reference fluorophore like RFP, and is not an inherent property of the organism. Please reword and clarify.

“while the aforementioned mapping methods used in C. Elegans can accommodate moderate changes in the total number of neurons between frames due to missing or spurious detections (counting noise), the intermittent and sparse detectability of neurons in calcium imaging definitely hinders the applicability of mapping methods to Hydra [7]”

**Answer:**  We replaced “*the intermittent and sparse detectability of neurons in calcium imaging definitely hinders the applicability of mapping methods to Hydra [7]*” with “*the intermittent and sparse detectability of neurons in calcium imaging, without a reference fluorophore like calcium-insensitive red fluorescent proteins (RFP) [10], definitely hinders the applicability of mapping methods.*”

• Subsection “4 - Validation of EMC^2”: “We used the first 250 frames” p.13 The authors should specify in the main text the duration in seconds of these 250 frames (Perhaps 25 seconds?) This information is important for the reader to interpret the method’s performance.

**Answer:**  We specified that 250 frames correspond to 25 seconds.

• In the abstract the authors write: “Our results prove…”. The statement of proof is unnecessarily, and in my opinion, inappropriately strong. Best usage would be to reserve proofs for mathematical proofs. Please revise.

**Answer:**  We replaced “Our results prove..” with “Our results show..”
